# Supplementary material for: Getting More Out of Biomedical Documents with GATE's Full Lifecycle Open Source Text Analytics
Source: PLoS Comput Biol. 2013 Feb 7;9(2):e1002854. doi: 10.1371/journal.pcbi.1002854 (PMC3567135; doi:10.1371/journal.pcbi.1002854)
Supplement: Dataset S1 — GATE software. Dataset S1 bundled with this paper contains a distribution of GATE (or see http://gate.ac.uk/download/). (TGZ) [file pcbi.1002854.s001.tgz › plos-gate/doc/index.html]

GATE Developer Page


Developers:   
| docs | licencing | contributing |   
| requirements | philosophy |

## Developer Documentation

- The User Guide includes developer documentation - that's the first place to look.
- A partial list of plugins for GATE.
- The last stable release (version 7.0 build 4195 - February 8^th^ 2012): JavaDoc (class names link to the HTML of the source code).
- Last night's build JavaDoc (class names link to the HTML of the source code).
- How to install GATE.
- How to report creepy-crawlies (bugs).
- Code style (**mandatory** for changes to GATE itself).
- Details of nightly build and test results.
- Release notes.
- What version of GATE this documentation set is for.

## Embedding GATE in other software systems

GATE Developer and GATE Embedded are licenced under the GNU Lesser General Public License, version
3 of June 29th 2007 (referred to as "The GNU License" below). (We also use lots of 3rd-party tools which are licenced separately - see the library licences list.)

Under the terms of the GNU License, the source code of a system that uses GATE as a library consitutes "a work that uses the library" and is therefore exempt from the terms of the license. Executables of embedding systems (that are linked with GATE) constitute "an executable that is derivative of the library" and are subject to the terms of clause 6 of the GNU License. This clause requires that if these executables are redistributed they must include a copy of GATE (both binary and source), a copy of the GNU License attached to that copy, and give notice that GATE used within it, including this information in any display of copyright notices given by the embedding system.

If you modify GATE, you must: clearly mark what modifications you made (e.g. in the headers of the files you changed), and you must make those modifications available free to anyone who wants them. This **does not apply** to work that is identifiably not part of the library, such as components you may write that are loaded with GATE at runtime. The license does not affect your rights to this work, only to modifications to the core GATE system itself.

Basically, you can use GATE in another system as you like. The only restriction is that if you distribute binaries that incorporate GATE, you must also include copies of GATE, and you must acknowledge your use of our system.

## Contributing

We welcome input to the development process of GATE. The code is hosted on SourceForge, providing anonymous Subversion access. We're happy to give committer privileges to anyone contributing good code to the project. We also make the current version available nightly on the continuous integration site.

**See also the community page.**

| gate home |
